# Development and validation of a nomogram to predict the risk factors of major complications after radical rectal cancer surgery

**DOI:** 10.3389/fonc.2024.1380535

**Published:** 2024-03-21

**Authors:** Quan Lv, Ye Yuan, Shu-Pei Qu, Yu-Hang Diao, Zhan-Xiang Hai, Zheng Xiang, Dong Peng

**Affiliations:** Department of Gastrointestinal Surgery, The First Affiliated Hospital of Chongqing Medical University, Chongqing, China

**Keywords:** rectal cancer, surgery, complications, nomogram, risk factors

## Abstract

**Purpose:**

The aim of this study was to establish a validated nomogram to predict risk factors for major post-operative complications in patients with rectal cancer (RC) by analyzing the factors contributing to major post-operative complications in RC patients.

**Methods:**

We retrospectively collected baseline and surgical information on patients who underwent RC surgery between December 2012 and December 2022 at a single-center teaching hospital. The entire cohort was randomly divided into two subsets (60% of the data for development, 40% for validation). Independent risk factors for major post-operative complications were identified using multivariate logistic regression analyses, and predictive models were developed. Area under the curve (AUC) was calculated using receiver operating characteristic curve (ROC) to assess predictive probability, calibration curves were plotted to compare the predicted probability of the nomogram with the actual probability, and the clinical efficacy of the nomogram was assessed using decision curve analysis (DCA).

**Results:**

Our study included 3151 patients who underwent radical surgery for RC, including 1892 in the development set and 1259 in the validation set. Forty (2.1%) patients in the development set and 26 (2.1%) patients in the validation set experienced major post-operative complications. Through multivariate logistic regression analysis, age (p<0.01, OR=1.044, 95% CI=1.016-1.074), pre-operative albumin (p<0.01, OR=0.913, 95% CI=0.866-0.964), and open surgery (p<0.01, OR=2.461, 95% CI=1.284-4.761) were identified as independent risk factors for major post-operative complications in RC, and a nomogram prediction model was established. The AUC of the ROC plot for the development set was 0.7161 (95% Cl=0.6397-0.7924), and the AUC of the ROC plot for the validation set was 0.7191 (95% CI=0.6182-0.8199). The predicted probabilities in the calibration curves were highly consistent with the actual probabilities, which indicated that the prediction model had good predictive ability. The DCA also confirmed the good clinical performance of the nomogram.

**Conclusion:**

In this study, a validated nomogram containing three predictors was created to identify risk factors for major complications after radical RC surgery. Due to its accuracy and convenience, it could contribute to personalized management of patients in the perioperative period.

## Introduction

Colorectal cancer is one of the most prevalent cancers in the world and is a serious threat to human health, with an estimated 1.9 million new cases and 935,000 deaths in 2020 (1). In recent years, with the rapid development of laparoscopic instruments and techniques, transabdominal low anterior resection (LAR) combined with total mesorectal excision (TME) has become the standard approach for the treatment of low and intermediate rectal cancer (RC). Laparoscopic rectal surgery (LRS) has been widely used for the treatment of RC because of its low trauma rate and fast recovery (2). A clear surgical field and full exposure of anatomical structures enabled LRS to achieve radical resection of RC, reduce surgical trauma, and improve the post-operative quality of life (3). However, post-operative complications remained a major concern. Previous studies have reported that the incidence of post-operative complications in RC was 20%-30%, the incidence of serious complications was 5%-12%, and the mortality rate was approximately 2% (4, 5). Anastomotic leakage (AL), a common serious complication after radical resection for RC, had an incidence of 2.4% to 27.0% and a mortality rate of 18% (6–8). These complications and bowel dysfunction might affect the patients’ quality of life and long-term prognosis.

In recent years, anastomotic devices and surgical techniques have improved considerably, however, the incidence of complications has not decreased significantly (9–12). Many previous randomized controlled studies have explored the risk factors for post-operative complications in RC, including age (13), pre-operative albumin (14), pre-operative neoadjuvant therapy (15) and body mass index (BMI) (16). Tumor-related factors included tumor size and the distance of the tumor from the anal verge (17, 18). Surgery-related factors included the duration of surgery and intraoperative blood loss (19). There were conflicting reports on the risk factors for complications after radical RC.

The Clavien-Dindo system has been widely used to classify post-operative complications. Clavien-Dindo III-IV complications requiring re-operation and endoscopic or radiological intervention were defined as serious complications (20), which always led to catastrophic consequences such as organ failure or even death, as well as high medical costs.

Therefore, the aim of this study was to establish a validated nomogram to predict risk factors for major post-operative complications in patients with RC by analyzing the factors contributing to post-operative complications in RC patients, and to provide a reference point for the prevention and treatment of post-operative complications for RC and provides timely and effective interventions in the peri-operative period.

## Materials and methods

### Patient selection

We retrospectively collected baseline and surgical information on patients who underwent radical RC surgery between December 2012 and December 2022 at a single-center teaching hospital. The inclusion criteria were that patients with a pathologically confirmed preoperative diagnosis of rectal malignancy who underwent radical surgery for RC. The exclusion criteria were as follows: 1. patients who underwent RC after recurrence; 2. patients with metastatic RC; 3. patients who underwent emergency surgery, including bowel obstruction and bleeding; and 4. patients with incomplete baseline or surgical information. Ultimately, 3151 patients with complete information were finally enrolled in the study, who were randomly assigned in a 6:4 ratio to the development set (n=1892) and validation set (n=1259) based on computer-generated random numbers.

This study was approved by the Ethics Committee of the First Affiliated Hospital of Chongqing Medical University (K2024-002-01). It complied with the principles of medical ethics and the Declaration of Helsinki, and all patients participating in the study signed an informed consent form.

### Data elements

We retrospectively collected baseline and surgical information of the patients. The baseline information included age, sex, BMI, smoking and drinking history, previous abdominal surgery (PAS), and preoperative comorbidities. Clinical information included preoperative albumin and hemoglobin, tumor stage, and tumor size. Surgical information included surgical methods, surgical time, blood loss, and major post-operative complications. The pre-operative comorbidities included hypertension, type 2 diabetes mellitus (T2DM), and chronic heart disease (CHD).

### Surgery management

All patients who underwent radical resection according to the guidelines of the Chinese Society of Clinical Oncology (CSCO) for colorectal cancer, that’s total mesorectal excision or complete mesocolic excision, and the post-operative pathology was confirmed R0 resection.

### Definition

Tumors were staged according to the 8th edition of the American Joint Committee on Cancer (AJCC) guidelines and were classified as stages I-IV (21).

Major post-operative complications within 30 days of surgery were assessed using the Clavien-Dindo scale (22). Clavien-Dindo III/IV complications requiring surgical, endoscopic, or radiological intervention were defined as major complications.

### Statistical analysis

All data in this study were processed using SPSS (version 22.0) and R (version 4.1.2). Continuous variables that followed a normal distribution were expressed as mean ± standard deviation (SD) and comparisons were made using the t-test; categorical variables were expressed as numbers and percentages, and chi-squared or Fisher’s exact test was used. Univariate logistic regression analysis was performed on all variables, and variables with P<0.05 were considered potential risk factors for the occurrence of major post-operative complications in RC patients. The screened potential risk factors were subjected to multivariate logistic regression analysis to identify independent predictors of complications after RC surgery. Finally, multivariate logistic regression analysis included variables with P<0.05, and a nomogram was created to predict the risk of major post-operative complications in RC.

The predictive models were evaluated in three ways. First, the predictive value of the risk factors was verified using receiver operating characteristic curve (ROC), and the performance of the nomogram was assessed by calculating the area under the curve (AUC) for the development and validation sets. The AUCs ranged from 0 and 1, with 1 indicating perfect agreement, 0.5 indicating no better than chance, and greater than 0.7 indicating that the model had relatively good predictive power (23, 24). Second, prediction curves were plotted to test the calibration of major post-operative complication risk map, and the predicted and actual probabilities of the nomogram for the development and validation sets were analyzed and compared, using the 45-degree line as the perfect model with 100% accuracy (25). Finally, decision curve analysis (DCA) was used to analyze the net benefits of the development and validation sets based on different threshold probabilities to determine the clinical applicability of the nomogram (26, 27).

## Results

### Baseline information

Based on the above inclusion and exclusion criteria, 3151 patients who underwent radical RC surgery were included in this study. This included 1892 patients in the development set and 1259 patients in the validation set. Forty (2.1%) patients in the development set and 26 (2.1%) patients in the validation set experienced major post-operative complications. Baseline information was comparable between the two groups (P>0.05) (**Table 1**).

**Table 1:** Baseline information between the development and validation cohorts.

Characteristics	Development (1892)	Validation (1259)	P value
Age, year	63.5 ± 12.8	63.5 ± 12.8	0.881
Sex			0.438
Male	1054 (55.7%)	719 (57.1%)	
Female	838 (44.3%)	540 (42.9%)	
BMI, kg/m^2^	22.5 ± 3.1	22.5 ± 3.4	0.912
Smoking	685 (36.2%)	470 (37.3%)	0.521
Drinking	570 (30.1%)	376 (29.9%)	0.875
Hypertension	474 (25.1%)	341 (27.1%)	0.202
T2DM	231 (12.2%)	146 (11.6%)	0.604
CHD	94 (5.0%)	65 (5.2%)	0.807
PAS	531 (28.1%)	347 (27.6%)	0.757
Albumin, g/L	38.8 ± 5.7	38.7 ± 5.9	0.714
Hemoglobin, g/L	113.7 ± 26.4	113.9 ± 25.6	0.825
TNM stage			0.769
I	257 (13.6%)	160 (12.7%)	
II	939 (49.6%)	628 (49.9%)	
III	696 (36.8%)	471 (37.4%)	
Tumor size			0.077
< 5cm	943 (49.8%)	587 (46.6%)	
≥ 5cm	949 (50.2%)	672 (53.4%)	
Surgical methods			0.850
Open	378 (20.0%)	255 (20.3%)	
Laparoscopic	1514 (80.0%)	1004 (79.7%)	
Surgical time, min	214.9 ± 79.1	218.5 ± 79.2	0.217
Blood loss, mL	98.7 ± 137.3	107.9 ± 190.7	0.114
Major complications	40 (2.1%)	26 (2.1%)	0.925

Variables are expressed as the mean ± SD, n (%), *P-value <0.05.

T2DM, type 2 diabetes mellitus; BMI, body mass index; CHD, chronic heart disease; PAS, previous abdominal surgery.

### Nomogram variable screening

Univariate and multivariate logistic regression analyses, including baseline, and surgical information, were performed to identify the risk factors influencing the occurrence of major post-operative complications in RC. The results of univariate logistic regression analysis showed that age (p<0.01, OR=1.044, 95% CI=1.016-1.074), preoperative albumin (p<0.01, OR=0.913, 95% CI=0.866-0.964), and open surgery (p<0.01, OR=2.461, 95% CI=1.284-4.761) were potential risk factors for major post-operative complications of RC. Further multivariate logistic regression analysis of the three potential risk factors showed that age (p=0.023, OR=1.033, 95% CI=1.005-1.062), pre-operative albumin (p=0.032, OR=0.940, 95% CI=0.888-0.995), and open surgery (p=0.049, OR=1.992, 95% CI=1.003-3.956) were independent risk factors for the occurrence of major post-operative complications in RC (**Table 2**).

**Table 2:** Univariate and multivariate logistic regression analysis of the major complications.

Risk factors	Univariate logistic regression analysis		Multivariate logistic regression analysis	
	OR (95% CI)	P value	OR (95% CI)	P value
Age, year	1.044 (1.016-1.074)	<0.01*	1.033 (1.005-1.062)	0.023*
Sex (female/male)	0.672 (0.349-1.295)	0.235		
BMI, Kg/m^2^	0.983 (0.889-1.087)	0.742		
Smoking (yes/no)	1.059 (0.554-2.022)	0.863		
Drinking (yes/no)	1.119 (0.573-2.186)	0.741		
Hypertension (yes/no)	1.821 (0.952-3.484)	0.070		
T2DM (yes/no)	1.028 (0.399-2.651)	0.955		
CHD (yes/no)	1.569 (0.475-5.185)	0.460		
PAS (yes/no)	0.972 (0.482-1.959)	0.936		
Albumin, g/L	0.913 (0.866-0.964)	<0.01*	0.940 (0.888-0.995)	0.032*
Hemoglobin, g/L	0.997 (0.986-1.009)	0.673		
Tumor stage (III/II/I)	1.242 (0.769-2.005)	0.376		
Tumor size (≥ 5/<5), cm	0.994 (0.531-1.859)	0.984		
Surgical methods (open/laparoscopic)	2.461 (1.284-4.761)	<0.01*	1.992 (1.003-3.956)	0.049*
Surgical time, min	0.997 (0.993-1.002)	0.268		
Blood loss, mL	1.000 (0.998-1.002)	0.777		

*P-value <0.05.

OR, Odds ratio; CI, confidence interval; BMI, body mass index; T2DM, type 2 diabetes mellitus; BMI, body mass index; CHD, chronic heart disease; PAS, previous abdominal surgery.

### Development of a nomogram to predict the occurrence of major complications after RC surgery

**Figure 1:**
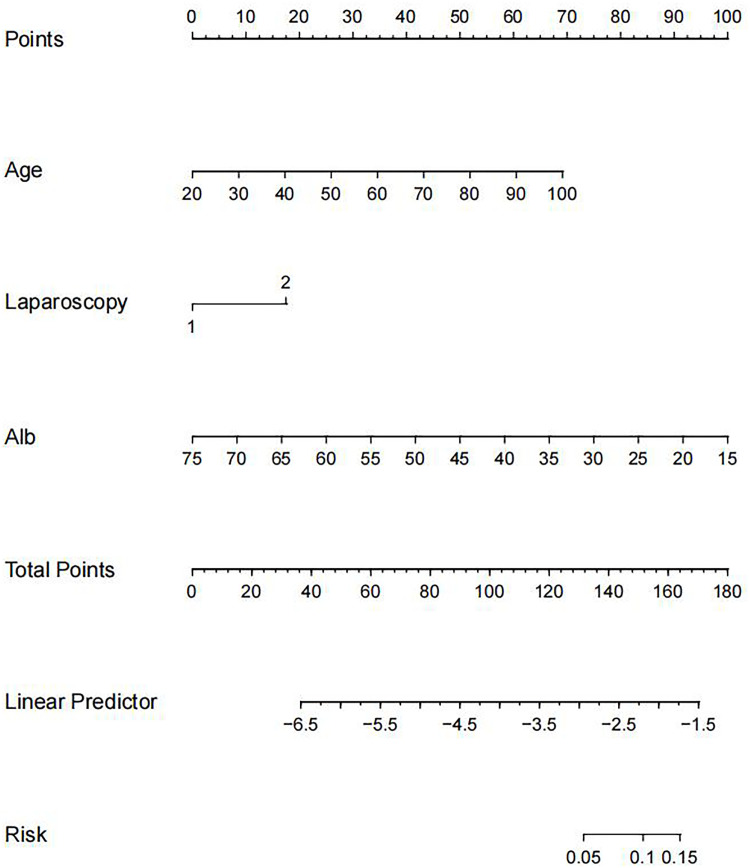
Nomogram for predicting the risk of major postoperative complications after RC surgery. RC, rectal cancer.

Using the three independent risk factors identified by the multivariate logistic regression analysis, a nomogram model was constructed to predict the risk of major post-operative complications in RC patients. As shown in **Figure 1**, the corresponding scores for each factor were derived from the patients’ own actual situation, and the three scores were added to derive the total score. The final predicted risk of major post-operative complications was the probability corresponding to the patient’s individual total score.

### Validation of a nomogram for predicting major complications after RC surgery

**Figure 2:**
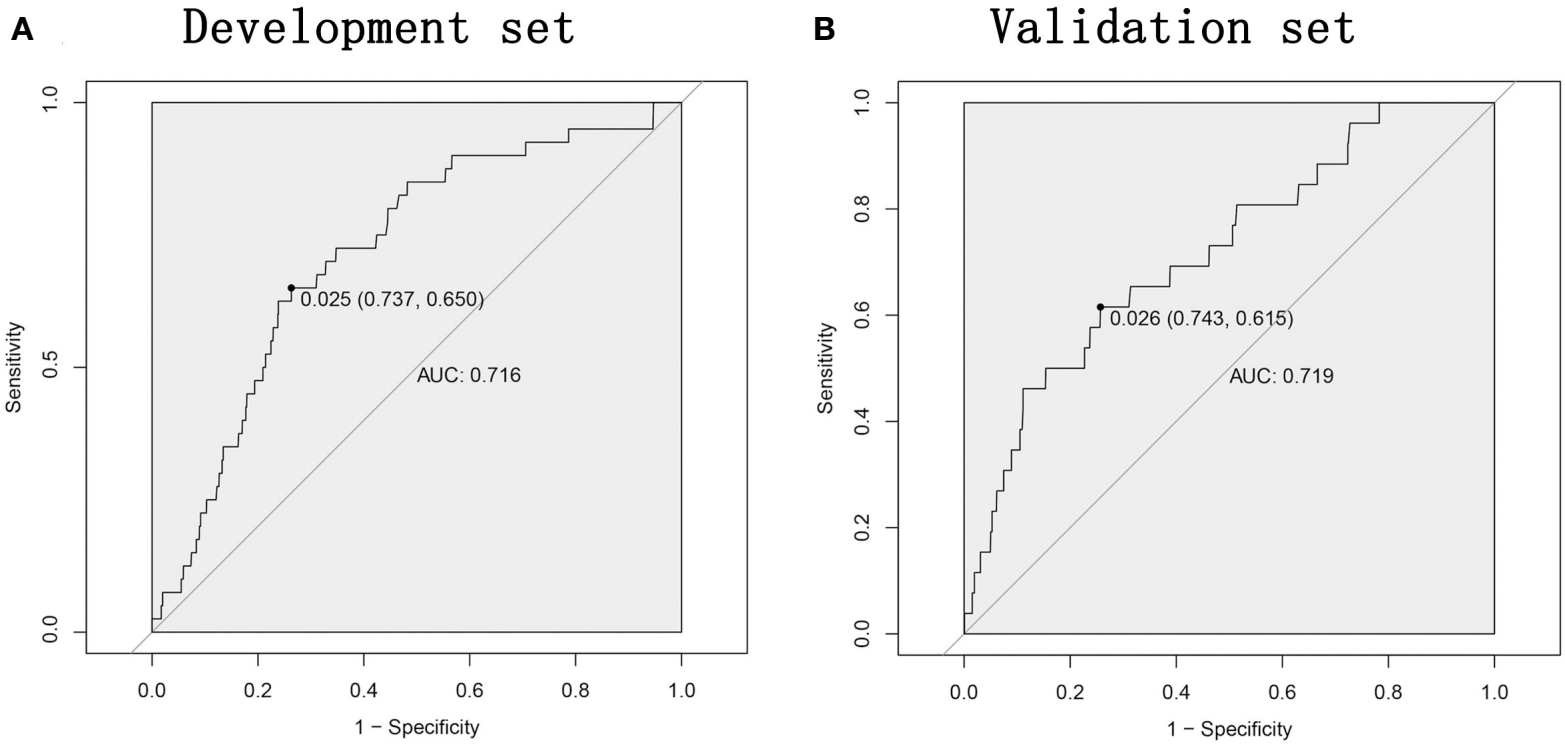
The nomogram model predicts the receiver operating characteristic ROC curve for major complications after rectal cancer surgery. **(A)** The area under the curve of the development set is 0.7161. **(B)** The area under the curve of the validation set is 0.7191. ROC, receiver operating characteristic; AUC, area under the curve.

**Figure 3:**
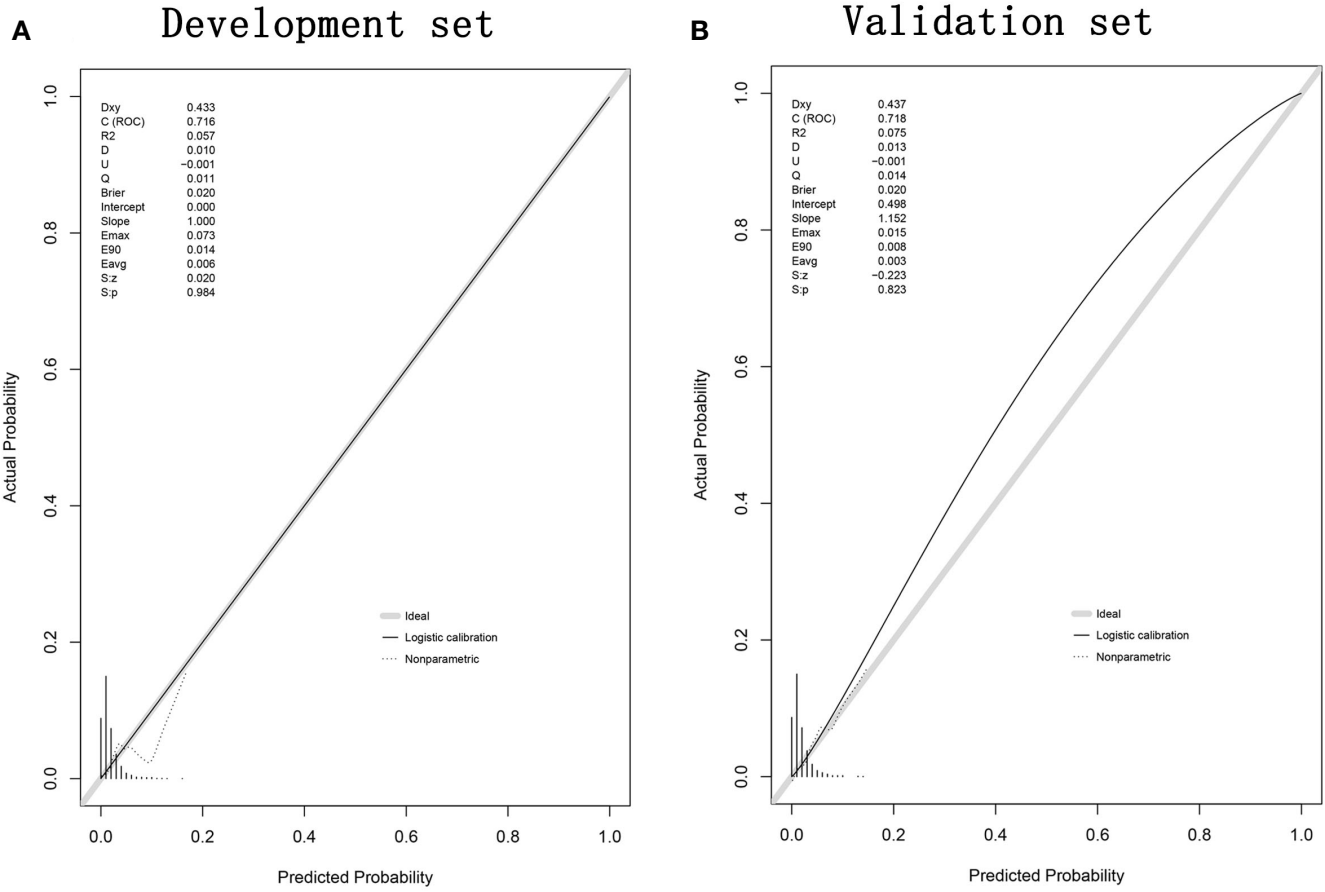
Calibration curves for development set **(A)** and validation set **(B)** nomograms.

**Figure 4:**
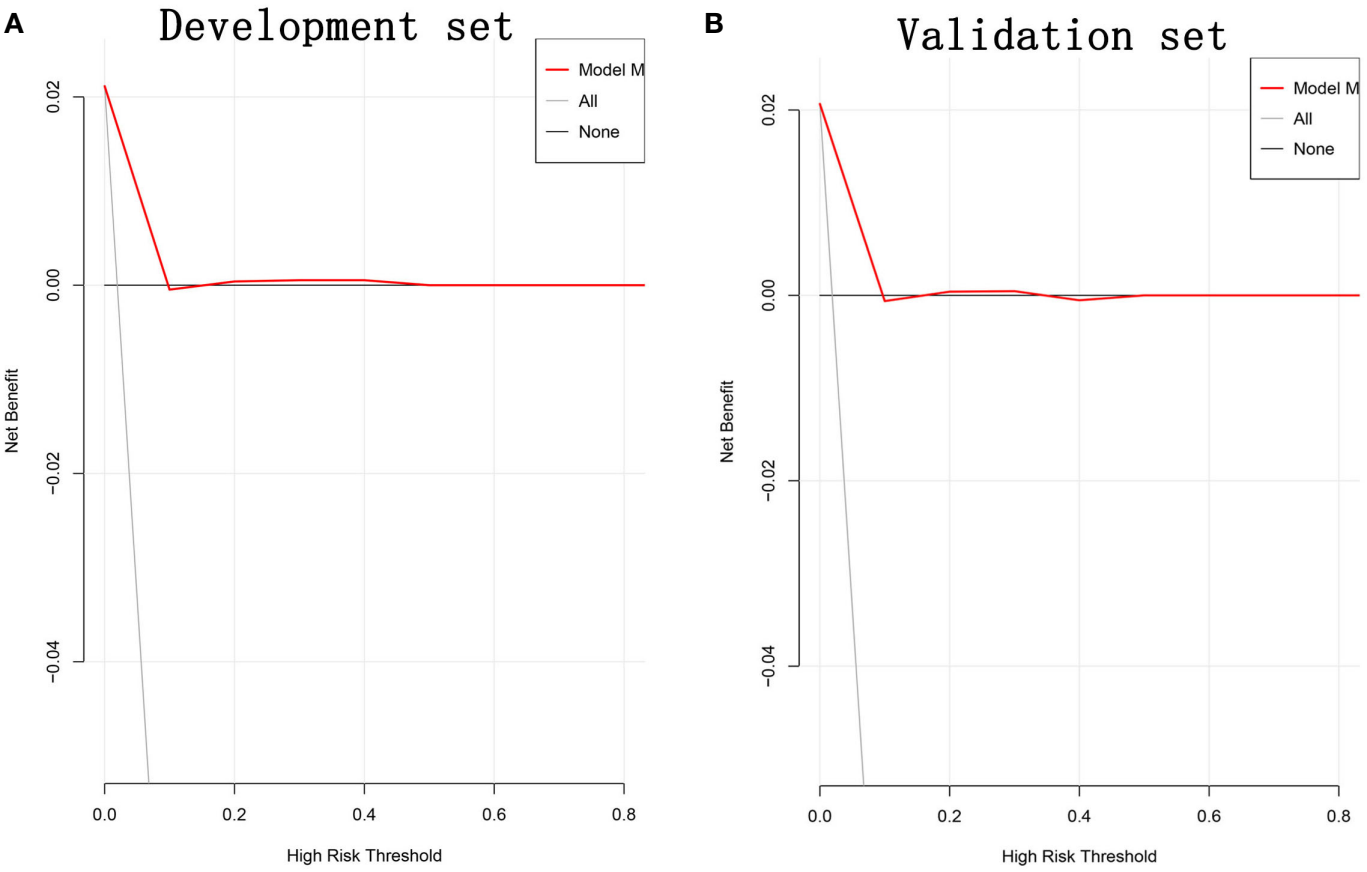
DCA for development set **(A)** and validation set **(B)**. DCA, decision curve analysis.

The ROC curve was used to assess the predictive accuracy of the nomogram. The results showed that the area under the ROC curve for the development set was 0.7161 (95% Cl=0.6397-0.7924), and that of the validation set was 0.7191 (95% CI=0.6182-0.8199). (**Figure 2**) The calibration curve showed a high degree of agreement between the predicted and observed results of the nomogram model constructed in this study. (**Figure 3**) Finally, DCA was used to evaluate the clinical application value of the prediction model, as shown in **Figure 4**.

## Discussion

Our study included 3151 patients who underwent radical surgery for RC, including 1892 in the development set and 1259 in the validation set. Forty (2.1%) patients in the development set and 26 (2.1%) patients in the validation set experienced major post-operative complications. Multivariate logistic regression analysis showed that age, pre-operative albumin, and open surgery were independent risk factors for the major post-operative complications of RC. Based on the three independent risk factors, we constructed a nomogram model to predict the risk factors of major post-operative complications in patients for RC.

In recent years, anastomosis and surgical techniques have developed considerably, but the incidence of post-operative complications in RC has not been significantly reduced (9–12). Several previous studies have shown that post-operative complications affected the prognosis of RC (28–30). Therefore, it was necessary to develop a predictive nomogram for major post-operative complications in RC. In our study, 66 (2.1%) patients had major post-operative complications, which was significantly lower than those reported in previous studies (31). This might be related to the definition of major post-operative complications.

The results of this study suggested that age was an independent risk factor for major post-operative complications for RC. This was like previous studies (32). Surgery-related comorbidities, including cardiovascular and pulmonary diseases, oncological anemia, and liver or kidney disease were more common in elderly patients (33, 34). In addition, neurological or psychological disorders was often prevalent in elderly patients (35). Previous studies found that elderly patients with limited baseline performance status (defined by Eastern Cooperative Group performance statuses 2-4, abnormalities in activities of daily living and instrumental activities of daily living) were less likely to tolerate the procedure and had worse outcomes than younger patients (36).

Studies have shown that low pre-operative albumin negatively affected wound healing and disease severity (37). In RC surgery, low pre-operative albumin levels significantly increased the incidence of post-operative complications (38, 39). Albumin has been reported to play a variety of roles, including stabilization of cell growth, DNA replication, maintenance of sex hormone balance and modulation of systemic inflammation (40). In addition, albumin levels were widely used as a variety of prognostic indicators, including the prognostic nutritional index (41), the systemic inflammation index (42) and played an important role in maintaining colloid osmolality, scavenging free radicals, and altering capillary membrane permeability (43). The important physiological function of serum albumin might be an important reason why albumin was prediction of serious post-operative complications.

The current study suggested that open surgery was an independent risk factor for major post-operative complications for RC. Compared with open surgery, laparoscopic rectal surgery was widely used in the treatment of RC because of its less trauma and faster recovery (2). Clear surgical vision and full exposure of anatomical structures enable laparoscopic RC surgery to achieve radical resection, reduce surgical trauma, and improve post-operative quality of life (3). Which had similar results with previous studies (44, 45).

The application of these three risk predictors to our model was crucial. Despite the good performance of our nomogram, this study had some limitations. First, this was a single-center retrospective study. Second, the pre-operative baseline and clinical information included were imperfect, including pre-operative neoadjuvant chemo-radiotherapy, the relationship to the rectal mesenteric fascia, the final distance from the anal verge, and the long-term efficacy of RC surgery. Third, in this study, a subset of patients underwent protective ileostomies. The results might be affected by the protective ileostomy. Fourth, because this study focused on post-operative major complications in RC, it was lacking types of surgery. Finally, the nomogram prediction model established in this study has not been internally validated, and we will continue to collect clinical data from relevant patients to further improve the internal validation. In the future, we hope that our study will be a joint effort of multiple centers to collect as many variables as possible and to continuously test and revise the prediction model in clinical practice.

In this study, a validated nomogram containing three predictors was created to identify risk factors for major complications after radical RC surgery. Due to its accuracy and convenience, it could contribute to personalized management of patients in the perioperative period.

## Data availability statement

The raw data supporting the conclusions of this article will be made available by the authors, without undue reservation.

## Ethics statement

This study was approved by the Ethics Committee of the First Affiliated Hospital of Chongqing Medical University (K2024-002-01). It complied with the principles of medical ethics and the Declaration of Helsinki, and all patients participating in the study signed an informed consent form. The studies were conducted in accordance with the local legislation and institutional requirements. The participants provided their written informed consent to participate in this study.

## Author contributions

QL: Conceptualization, Data curation, Resources, Validation, Writing – original draft. YY: Resources, Writing – original draft. S-PQ: Data curation, Formal analysis, Investigation, Validation, Writing – original draft. Y-HD: Conceptualization, Investigation, Methodology, Resources, Writing – original draft. Z-XH: Data curation, Investigation, Project administration, Writing – original draft. ZX: Data curation, Methodology, Validation, Writing – review & editing. DP: Data curation, Funding acquisition, Methodology, Writing – review & editing.

## References

[1] Sung H Ferlay J Siegel RL Laversanne M Soerjomataram I Jemal A. Global cancer statistics 2020: GLOBOCAN estimates of incidence and mortality worldwide for 36 cancers in 185 countries. CA Cancer J Clin. (2021) 71:209–49. doi: 10.3322/caac.21660 33538338

[2] van der Pas MH Haglind E Cuesta MA Fürst A Lacy AM Hop WC. COlorectal cancer Laparoscopic or Open Resection II (COLOR II) Study Group. Laparoscopic versus open surgery for rectal cancer (COLOR II): short-term outcomes of a randomised, phase 3 trial. Lancet Oncol. (2013) 14:210–8. doi: 10.1016/S1470-2045(13)70016-0 23395398

[3] Akiyoshi T. Technical feasibility of laparoscopic extended surgery beyond total mesorectal excision for primary or recurrent rectal cancer. World J Gastroenterol. (2016) 22:718–26. doi: 10.3748/wjg.v22.i2.718 PMC4716071 26811619

[4] Paun BC Cassie S MacLean AR Dixon E Buie WD. Postoperative complications following surgery for rectal cancer. Ann Surg. (2010) 251:807–18. doi: 10.1097/SLA.0b013e3181dae4ed 20395841

[5] McSorley ST Horgan PG McMillan DC. The impact of the type and severity of postoperative complications on long-term outcomes following surgery for colorectal cancer: A systematic review and meta-analysis. Crit Rev Oncol Hematol. (2016) 97:168–77. doi: 10.1016/j.critrevonc.2015.08.013 26330375

[6] Matsubara N Miyata H Gotoh M Tomita N Baba H Kimura W. Mortality after common rectal surgery in Japan: a study on low anterior resection from a newly established nationwide large-scale clinical database. Dis Colon Rectum. (2014) 57:1075–81. doi: 10.1097/DCR.0000000000000176 25101603

[7] Matthiessen P Hallböök O Rutegård J Simert G Sjödahl R. Defunctioning stoma reduces symptomatic anastomotic leakage after low anterior resection of the rectum for cancer: a randomized multicenter trial. Ann Surg. (2007) 246:207–14. doi: 10.1097/SLA.0b013e3180603024 PMC1933561 17667498

[8] Liu XR Liu F Zhang W Peng D. The aortic calcification is a risk factor for colorectal anastomotic leakage. Updates Surg. (2023) 75:1857–65. doi: 10.1007/s13304-023-01630-4 37594659

[9] Arnarson Ö Butt-Tuna S Syk I. Postoperative complications following colonic resection for cancer are associated with impaired long-term survival. Color Dis. (2019) 21:805–15. doi: 10.1111/codi.14613 30884061

[10] Planellas P Farrés R Cornejo L Rodríguez-Hermosa JI Pigem A Timoteo A. Randomized clinical trial comparing side to end vs end to end techniques for colorectal anastomosis. Int J Surg. (2020) 83:220–9. doi: 10.1016/j.ijsu.2020.09.039 33038521

[11] Zeman M Czarnecki M Chmielik E Idasiak A Skałba W Strączyński M. The assessment of risk factors for long-term survival outcome in ypN0 patients with rectal cancer after neoadjuvant therapy and radical anterior resection. World J Surg Oncol. (2021) 19:154. doi: 10.1186/s12957-021-02262-x 34020673 PMC8140444

[12] Chen TY Wiltink LM Nout RA Meershoek-Klein Kranenbarg E Laurberg S Marijnen CA. Bowel function 14 years after preoperative short-course radiotherapy and total mesorectal excision for rectal cancer: report of a multicenter randomized trial. Clin Colorectal Cancer. (2015) 14:106–14. doi: 10.1016/j.clcc.2014.12.007 25677122

[13] Guo F Sun Z Wang Z Gao J Pan J Zhang Q. Nomogram for predicting prolonged postoperative ileus after laparoscopic low anterior resection for rectal cancer. World J Surg Oncol. (2023) 21:380. doi: 10.1186/s12957-023-03265-6 38082330 PMC10712154

[14] Lohsiriwat V Lohsiriwat D Boonnuch W Chinswangwatanakul V Akaraviputh T Lert-Akayamanee N. Pre-operative hypoalbuminemia is a major risk factor for postoperative complications following rectal cancer surgery. World J Gastroenterol. (2008) 14:1248–51. doi: 10.3748/wjg.14.1248 PMC2690674 18300352

[15] Yang J Luo Y Tian T Dong P Fu Z. Effects of neoadjuvant radiotherapy on postoperative complications in rectal cancer: A meta-analysis. J Oncol. (2022) 2022:8197701. doi: 10.1155/2022/8197701 35035483 PMC8754670

[16] Alqarni A Aljehaiman F Almousa SA Almarshad SA Alrzouq FK. The relationship between BMI and postoperative complications among colorectal cancer patients undergoing surgery. Cureus. (2023) 15:e48715. doi: 10.7759/cureus.48715 38094533 PMC10716719

[17] Wang K Li M Liu R Ji Y Yan J. Analysis of Risk Factors for Anastomotic Leakage After Laparoscopic Anterior Resection of Rectal Cancer and Construction of a Nomogram Prediction Model. Cancer Manag Res. (2022) 14:2243–52. doi: 10.2147/CMAR.S364875 PMC9343466 35928989

[18] Tao W Liu F Cheng YX Zhang B Liu XY Zhang W. Comparison of postoperative outcome and prognosis among laparoscopic left colectomy and laparoscopic sigmoidectomy in sigmoid colon cancer patients: A propensity score matching study. Cancer Control. (2023) 30:10732748231210676. doi: 10.1177/10732748231210676 37982606 PMC10664434

[19] Lu SQ Chang XF Yang XD Yu DC Huang QG Wang F. [Establishment of a nomogram predicting risk factors of postoperative perineal wound complications after abdominoperineal resection for rectal cancer]. Zhonghua Wei Chang Wai Ke Za Zhi. (2019) 22:357–63. doi: 10.3760/cma.j.issn 31054550

[20] Dindo D Demartines N Clavien PA. Classification of surgical complications: a new proposal with evaluation in a cohort of 6336 patients and results of a survey. Ann Surg. (2004) 240:205–13. doi: 10.1097/01.sla.0000133083.54934.ae PMC1360123 15273542

[21] Amin MB Greene FL Edge SB Compton CC Gershenwald JE Brookland RK. The Eighth Edition AJCC Cancer Staging Manual: Continuing to build a bridge from a population-based to a more “personalized” approach to cancer staging. CA Cancer J Clin. (2017) 67:93–9. doi: 10.3322/caac.21388 28094848

[22] Bolliger M Kroehnert JA Molineus F Kandioler D Schindl M Riss P. Experiences with the standardized classification of surgical complications (Clavien-Dindo) in general surgery patients. Eur Surg. (2018) 50:256–61. doi: 10.1007/s10353-018-0551-z PMC6267508 30546385

[23] Liu Q Zhou Q Song M Zhao F Yang J Feng X. A nomogram for predicting the risk of sepsis in patients with acute cholangitis. J Int Med Res. (2020) 48:300060519866100. doi: 10.1177/0300060519866100 31429338 PMC7140205

[24] Yu P Kan R Meng X Wang Z Xiang Y Mao B. A nomogram for predicting the risk of CKD based on cardiometabolic risk factors. Int J Gen Med. (2023) 16:4143–54. doi: 10.2147/IJGM.S425122 PMC10503556 37720178

[25] Lin Z Li Y Wu J Zheng H Yang C. Nomogram for prediction of prolonged postoperative ileus after colorectal resection. BMC Cancer. (2022) 22:1273. doi: 10.1186/s12885-022-10377-x 36474177 PMC9724353

[26] Shi B Shen L Huang W Cai L Yang S Zhang Y. A nomogram for predicting surgical timing in neonates with necrotizing enterocolitis. J Clin Med. (2023) 12:3062. doi: 10.3390/jcm12093062 37176503 PMC10179100

[27] Huai J Ye X Ding J. Nomogram for the prediction of delayed colorectal post-polypectomy bleeding. Turk J Gastroenterol. (2021) 32:727–34. doi: 10.5152/tjg.2021.20842 PMC8975504 34609301

[28] Liu XY Li ZW Zhang B Liu F Zhang W Peng D. Effects of preoperative bicarbonate and lactate levels on short-term outcomes and prognosis in elderly patients with colorectal cancer. BMC Surg. (2023) 23:127. doi: 10.1186/s12893-023-02039-x 37189084 PMC10186757

[29] Nowakowski M Pisarska M Rubinkiewicz M Torbicz G Gajewska N Mizera M. Postoperative complications are associated with worse survival after laparoscopic surgery for non-metastatic colorectal cancer - interim analysis of 3-year overall survival. Wideochir Inne Tech Maloinwazyjne. (2018) 13:326–32. doi: 10.5114/wiitm.2018.76179 PMC6174179 30302145

[30] Liu XY Zhang B Kang B Cheng YX Yuan C Tao W. The effect of complications on oncological outcomes of colorectal cancer patients after primary surgery: A propensity score matching analysis. Front Oncol. (2022) 12:857062. doi: 10.3389/fonc.2022.857062 35719908 PMC9203956

[31] Liang LC Liu DL Liu SJ Hu L He YR Wan X. Risk factors for severe complications after laparoscopic surgery for T3 or T4 rectal cancer for Chinese patients: experience from a single center. Med Sci Monit. (2020) 26:e920604. doi: 10.12659/MSM.920604 32764534 PMC7433389

[32] Turri G Caliskan G Conti C Martinelli L De Giulio E Ruzzenente A. Impact of age and comorbidities on short- and long-term outcomes of patients undergoing surgery for colorectal cancer. Front Oncol. (2022) 12:959650. doi: 10.3389/fonc.2022.959650 36338732 PMC9633938

[33] Gröne J Kreis ME. Individualisierte Therapie des kolorektalen Karzinoms im hohen Alter [Personalized treatment of colorectal cancer in old age]. Chirurg. (2013) 84:305–9. doi: 10.1007/s00104-012-2452-1 23494055

[34] Forman DE Maurer MS Boyd C Brindis R Salive ME Horne FM. Multimorbidity in older adults with cardiovascular disease. J Am Coll Cardiol. (2018) 71:2149–61. doi: 10.1016/j.jacc.2018.03.022 PMC6028235 29747836

[35] Person H Keefer L. Psychological comorbidity in gastrointestinal diseases: Update on the brain-gut-microbiome axis. Prog Neuropsychopharmacol Biol Psychiatry. (2021) 107:110209. doi: 10.1016/j.pnpbp.2020.110209 33326819 PMC8382262

[36] Pope D Ramesh H Gennari R Corsini G Maffezzini M Hoekstra HJ. Pre-operative assessment of cancer in the elderly (PACE): a comprehensive assessment of underlying characteristics of elderly cancer patients prior to elective surgery. Surg Oncol. (2006) 15:189–97. doi: 10.1016/j.suronc.2007.04.009 17531743

[37] Sofić A Rašić I Halilović E Mujić A Muslić D. Is preoperative hypoproteinemia associated with colorectal cancer stage and postoperative complications? Med Glas (Zenica). (2021) 18:450–5. doi: 10.17392/1353-21 34190507

[38] Kang B Zhao ZQ Liu XY Cheng YX Tao W Wei ZQ. Effect of hypoalbuminemia on short-term outcomes after colorectal cancer surgery: A propensity score matching analysis. Front Nutr. (2022) 9:925086. doi: 10.3389/fnut.2022.925086 36105581 PMC9464913

[39] Liu ZJ Ge XL Ai SC Wang HK Sun F Chen L. Postoperative decrease of serum albumin predicts short-term complications in patients undergoing gastric cancer resection. World J Gastroenterol. (2017) 23:4978–85. doi: 10.3748/wjg.v23.i27.4978 PMC5526768 28785152

[40] Gupta D Lis CG. Pretreatment serum albumin as a predictor of cancer survival: a systematic review of the epidemiological literature. Nutr J. (2010) 9:69. doi: 10.1186/1475-2891-9-69 21176210 PMC3019132

[41] Tokunaga R Sakamoto Y Nakagawa S Miyamoto Y Yoshida N Oki E. Prognostic nutritional index predicts severe complications, recurrence, and poor prognosis in patients with colorectal cancer undergoing primary tumor resection. Dis Colon Rectum. (2015) 58:1048–57. doi: 10.1097/DCR.0000000000000458 26445177

[42] Hong T Shen D Chen X Cai D Wu X Hua D. A novel systematic inflammation related index is prognostic in curatively resected non-metastatic colorectal cancer. Am J Surg. (2018) 216:450–7. doi: 10.1016/j.amjsurg.2017.07.021 28743381

[43] Caraceni P O’Brien A Gines P. Long-term albumin treatment in patients with cirrhosis and ascites. J Hepatol. (2022) 76:1306–17. doi: 10.1016/j.jhep.2022.03.005 35589252

[44] Miyakawa T Michihata N Kumazawa R Matsui H Honda M Yasunaga H. Short-term surgical outcomes of laparoscopic and open surgery for rectal cancer: A nationwide retrospective analysis. Asian J Endosc Surg. (2023) 16:376–85. doi: 10.1111/ases.13166 36693819

[45] Jiang JB Jiang K Dai Y Wang RX Wu WZ Wang JJ. Laparoscopic versus open surgery for mid-low rectal cancer: a systematic review and meta-analysis on short- and long-term outcomes. J Gastrointest Surg. (2015) 19:1497–512. doi: 10.1007/s11605-015-2857-5 26040854

